# Detection and prevalence of *Anaplasma phagocytophilum *and *Rickettsia helvetica *in *Ixodes ricinus *ticks in seven study areas in Sweden

**DOI:** 10.1186/1756-3305-3-66

**Published:** 2010-08-04

**Authors:** Kristofer Severinsson, Thomas G Jaenson, John Pettersson, Kerstin Falk, Kenneth Nilsson

**Affiliations:** 1 Department of Medical Sciences, Clinical Bacteriology, Uppsala University, Uppsala, Sweden; 2 Medical Entomology Unit, Department of Systematic Biology, Evolutionary Biology Centre, Uppsala University, Uppsala, Sweden; 3 Department of Virology, Swedish Institute for Infectious Disease Control, Solna, Sweden; 4 Department of Medical Sciences, Infectious Diseases, Uppsala University, Uppsala, Sweden; 5 Department of Infectious Diseases, Falu County Hospital, Falun, Sweden; 6 Center of Clinical Research, Dalarna, Falun, Sweden

## Abstract

**Background:**

Tick-borne *Anaplasma phagocytophilum *and *Rickettsia *spp. are considered to be emerging human pathogens, but only limited data are available on their occurrence in Sweden. Two real-time PCR assays followed by nested PCR and sequence analysis were carried out to investigate the prevalence of *A. phagocytophilum *and spotted fever rickettsiae in ticks from seven areas in Sweden.

**Results:**

In 139 pooled samples, representing a total of 1245 *Ixodes ricinus *ticks (204 larvae, 963 nymphs, 38 males, 40 females), the overall positive mean infection prevalence was 1.3-15.0% for *A. phagocytophilum *and 1.5-17.3% for *R. helvetica. A. phagocytophilum *was only detected in nymphs (1.7-19.4%), whereas *R. helvetica *was detected in all three tick stages. Support for vertical and transstadial transmission was only obtained for *R. helvetica*. Both agents showed similar infection rates across study areas, although infection rates were greater in coastal areas.

**Conclusions:**

The results show that both pathogens occurred in all seven locations, indicating that they are prevalent in Sweden and should be considered etiological agents in patients recently bitten by ticks.

## Background

Most ticks found on humans and other large and medium-sized mammals in Sweden belong to the hard tick species *Ixodes ricinus*, which is a well-known important vector of several agents causing human disease such as *Borrelia burgdorferi *s.l., *Anaplasma *spp., *Rickettsia *spp. and the tick-borne encephalitis virus [1]. After a recent revision of the family Anaplasmataceae, *Ehrlichia equi, E. phagocytophila *and the human granulocytic ehrlichiosis (HGE) agent are now represented by the single species *Anaplasma phagocytophilum *[2]. HGE is considered to be an emerging tick-borne disease. *Ixodes *ticks are the vectors - in Europe the main vector is *I. ricinus *- and are believed to be maintained in Eurasia, mainly in a tick (*I. ricinus, I. persulcatus, I. trianguliceps*) and small mammal (*Myodes, Apodemus, Sorex*) cycle with humans only involved as incidental, dead-end hosts [3-11]. In North America, the white-footed mouse (*Peromyscus leucopus*) and white-tailed deer (*Odocoileus virginianus*) are considered the main vertebrate reservoirs [12,13]. The prevalence of *A. phagocytophilum *in a larger representative tick population in Sweden has not been studied previously. The organism is known to invade granulocytes of various mammalian species and causes febrile disease in ruminants, horses and dogs [2,14]. The importance of *A. phagocytophilum *as a human pathogen in Sweden is more uncertain, but serologic evidence of *A. phagocytophilum *infection has been found in southern Sweden, where about 28% of residents were found to be seropositive to HGE [15,16]. Among the cases reported, the disease usually presents nonspecific symptoms including fever, headache, chills, myalgia, arthralgia and hematological abnormalities [17,18].

The spotted fever rickettsia (SFR), *Rickettsia helvetica*, which has been recovered in Europe, Africa and Asia, is the only tick-transmitted *Rickettsia *reported from Sweden [19,20]. Previous initial studies of ticks in Sweden have shown a variable prevalence of 1.7-22.1%. We have recently also investigated the role of migratory birds in the spread of this rickettsia [19,21]. A handful of documented patients have presented a mild, self-limited disease with fever, headache and myalgia, but a more severe acute febrile clinical picture and also subacute meningitis have also been described [22-24]. Thus, increased research on the prevalence of rickettsiae in ticks is important. To obtain more data on the occurrence of these agents in Sweden, we investigated, using polymerase chain reaction (PCR) technology, the prevalence and genetic properties of *A. phagocytophilum *and *Rickettsia *spp. in larval, nymphal and adult *I. ricinus *ticks collected at seven different localities in southeastern and central Sweden and along the coast of northern Sweden.

## Results

### Tick infestation and study areas

Of the 139 pooled samples, representing 1245 ticks consisting of 204 larvae, 963 nymphs and 78 adults (38 males, 40 females), 16 (11.5%) were PCR positive for *A. phagocytophilum *and 19 (13.7%) for *R. helvetica*. All tested samples were pooled, which is why re-testing of individual ticks was not possible. Therefore, the prevalence rates are the minimal-maximum infection rates, assuming that only one or all tick specimens were positive in each positive pool. The resulting prevalence is therefore calculated as an approximate value of an interval resulting in a possible infection rate for *A. phagocytophilum *of 1.3-15.0% and for *R. helvetica *of 1.5-17.3%.

The number of pooled samples per study area and the number of tick stages, the number, and percent of positive samples and infection rates in each tick stage, and the total range (minimum-maximum) prevalence (%) for *A*. *phagocytophilum *and *R*. *helvetica*, respectively, and the differences found in a comparison of coastal and inland areas are shown in Tables 1, 2, 3 and 4.

**Table 1:** Number of ticks and samples (in brackets) per tick stage and area.

Area	Larvae	Nymph	Adult Male	Adult Female	Total
**Lidingö**	12 (2)	100 (10)	6 (1)	3 (2)	121 (15)
**Alsike**	25 (3)	175 (22)	15 (4)	19 (6)	234 (35)
**Torö**	33 (3)	175 (13)	7 (2)	3 (1)	218 (19)
**Bogesnd**	2 (1)	327 (23)	7 (3)	14 (3)	350 (30)
**Västerås**	87 (10)	30 (6)	2 (2)	1 (1)	120 (19)
**Lidköping**	45 (3)	105 (11)	1 (1)	0 (0)	151 (15)
**Norrbyskär**	0 (0)	51 (6)	0 (0)	0 (0)	51 (6)
**Total**	204 (22)	963 (91)	38 (13)	40 (13)	1245 (139)

**Table 2:** *Anaplasma phagocytophilum *Number and percent of positive samples, number of ticks in the positive samples and possible infection rate calculated as a minimum-maximum range descriptive of one or all of the ticks were positive in each positive pool.

Area	Larvae	Nymph	Adult Male	Adult Female	Total
**Lidingö**	0	1/(10)/10/(1-10)	0	0	1/(8.8)/10/(0.8-8.3)
**Alsike**	0	2/(9.1)/16/(1.1-9.1)	0	0	2/(5.7)/16/(0.9-6.8)
**Torö**	0	6/(46.1)/80/(3.4-45.7)	0	0	6/(31.5)/80/(2.8-36.7)
**Bogesund**	0	4/(17.4)/51/(1.2-16.6)	0	0	4/(13.3)/51/(1.1-14.6)
**Västerås**	0		0	0	0/(0)/19/NA
**Lidköpng**	0	2/(18.1)/20/(1.9-19-0)	0	0	2/(13.3)/20/(1.3-13.2)
**Norrbyskär**	0	1/(16)/10/(1.9-19.6)	0	0	1/(16.6)/10/(1.7-19.6)
**Total**	0	16/(17.5)/187/(1.7-19.4)	0	0	16/(11.5)/187/(1.3-15.0)

The total ranges are calculated per area, stage and for the whole material. S = sample, NA = not applicable, T = tick, % = percent

**Table 3:** *R. Helvetica *Number and percent of positive samples, number of ticks in the positive samples and possible infection rate calculated as a minimum-maximun range descriptive of one or all of the ticks were positive in each positive pool.

Area	Larvae	Nymph	Adult Male	Adult Female	Total
**Lidingö**	0	2/(20)/20/(2-20)	0	0	2/(13.3)/20/(2-16.5)
**Alsike**	0	1/(4.5)/6/(0.6-3.4)	1/(25)/4/(6.6-26)	0	2/(5.7)/10/(0.8-4.2)
**Torö**	1/(38)/15/(3-45)	5/(38)/75/(2.9-42)	0	0	6/(31)/90/(2.8-41.2)
**Bogesund**	0	0	0	0	0/(0)/30/(0)
**Västerås**	0	0	0	0	0/(0)/19/(0)
**Lidköping**	1/(33)/20/(2.2-44)	5/(45)/50/(4.8-47.6)	0	0	6/(40)/70/(4-47)
**Norrbyskär**	0	3/(50)/25/(5.8-49)	0	0	3/(50)/25/(5.8-49)
**Total**	2/(9.1)/35/(1.0-17.2)	16/(17.5)/176/(1.6-18)	1/(7.6)/4/(2.6-10)	0	19/(13.7)/215/1.5-17.3)

The total ranges are calculated per area, stage and for the whole material. For abbreviations, see Table 2

**Table 4:** Summary of total number (No) of positive samples (S)/(%) positive S/No of ticks in samples positive for *A. phagocytophilum *and *R. helvetica */(min-max infection rate %) for each tick stage and whole material from coastal (L+B+T+N) and inland (A+V+L) localities.

	Larvae	Nymph	Adult Male	Adult Female	TOTAL
**COASTAL AREAS**					
**Total: No T (No S)**	47 (6)	653 (52)	20 (6)	20 (6)	740 (70)
**No Ap pos**	0	12/(23.1)/151/(1.8-23.1)	0	0	13/(18.6)/152/(1.8-20.5)
**No Rh pos**	1/(16)/15/(2.1-31.9)	10/(19.2)/120/(1.5-18.4)	0	0	11/(15.7)/135/(1.5-18.2)
					
**INLAND**					
**Total: No T (No S)**	157/(16)	310/(39)	18 (7)	20 (7)	505 (69)
**No Ap pos**	0	4/(10.3)/35/(1.3-25.9)	0	0	5/(7,2)/42/(1.0-8.3)
**No Rh pos**	1/(6.3)/20/(0.6-12.7)	6/(15.4)/56/(1.9-18.1)	1/(14.3)/4/(5.5-22.2)	0	8/(11.5)/80/(1.6-15.8)

Rh = *R. helvetica*, Ap = *A. phagocytophilum*. For other abbreviations, see Table 2

The overall number and prevalence of positive samples for *A. phagocytophilum *(11.5%) (Table 2) and the total infection rate (1.3-15.0%) (Table 2) were not significantly different to those found for *R. helvetica *(13.7% and 1.5-17.3%, respectively) (Table 3). *A. phagocytophilum *was only detected in nymphs (prevalence 17.5%, total infection rate 1.7-19.4%), and no larvae or adults were found to be infected (Table 2). In contrast, *R. helvetica *was found in all three tick stages and at similar infection rates (Table 3).

The mean number of DNA copies in the positive samples ranged from 15 to 41,000 copies (mean = 7500 copies). There was no significant difference among tick stages in the number of copies in single infected ticks. Because we analysed pooled samples, the number of individual positive ticks in the positive pools is unknown. However, in 9 of 19 samples, the number of copies was less than 6000, which is indicative of a single or a few positive ticks per positive pool and prevalence at the low end of the range.

The infection rates for *A. phagocytophilum *and *R. helvetica *were comparable in the same study area (Table 2 and 3). However, assuming that 15% or less of the ticks were infected, as also evidenced by the number of copies, *A. phagocytophilum *was significantly more prevalent in the coastal areas (Fisher's exact test p < 0.05), unlike *R. helvetica*, which showed similar prevalences in coastal and inland areas (Table 4).

Five samples containing a total of 60 nymphs were positive for both agents. Whether this represents co-infection of the same tick or infection by different tick individuals is not possible to determine on the basis of the present material.

### Sequencing and species identification

The real-time PCR used has a documented high specificity for *A. phagocytophilum*, and not for other species within the genus *Anaplasma*. To verify this property, the products amplified in the real-time PCR, for 6 of the positive samples, were sequenced and showed 99-100% similarity with *A. phagocytophilum *for the msp2 gene [GenBank: FJ81284.1]. One of these samples was further investigated using an additional nested PCR assay, yielding a 850 bp-long fragment that was complete. Five of the 16 positive samples were also further investigated using an additional conventional PCR assay, yielding a 151 bp-long fragment of which a 90-100 bp fragment resulted in a complete sequence. The obtained fragments (850 and 151 bp) from both nested PCR assays represent the 16 S rRNA gene, where sequence analysis of these amplicon products showed 98-100% similarity with the corresponding gene sequences of *A. phagocytophilum *[GenBank: GU06489].

All 19 samples for SFR were re-run in the conventional and nested PCR assays and produced amplicons of partial regions of the 17-kDa and *omp*B genes, where sequence analysis of all samples showed 99-100% similarity with the corresponding gene sequences of *R. helvetica *(17 kDa, [GenBank: AF181036.1], *ompB*, [GenBank: AF123725.1]) and significant nucleotide differences from other spotted fever group rickettsiae found in neighbouring countries. For example, the partial sequences obtained showed 95% similarity with *R. monacensis *for the 17 kDa gene [GenBank: EF380355.1], and 97 % similarity with *R monacensis *[GenBank: EF380356.1], for the *omp*B gene, presenting a difference of 19 nt and 5 nt, respectively. The similarity with *R. slovaca *[GenBank: AF123723.2], was 97% for the *ompB gene *representing an 8 nt difference.

## Discussion

This is the first investigation to describe the distribution and prevalence of *A. phagocytophilum *and *R. helvetica *in different stages of *I. ricinus *ticks in inland and coastal areas of Sweden. In previous studies in Sweden, *A. phagocytophilum *was only reported in nymphs from migratory birds and on the western and southeastern coast of Sweden at prevalence rates of 6.6-8% [3,4]. Our findings of overall 11.5 % positive tick samples (range 1.3-15%) represent positive nymph samples (infection range 1.7-19.4%). This is a prevalence similar to estimates from Denmark, where 14.5% of nymphs were positive, and from Poland, where the prevalence in *I. ricinus *ranged from 0-27.6% [6,7]. None of the adult tick samples (n = 78 ticks) were positive, compared to Denmark where the infection ratio of adult ticks was up to 40.5%.

It has been reported, however, that the infection prevalence for *A. phagocytophilum *may show great year-to-year variation, which is why prevalence across several years should be studied to obtain more reliable results for all stages [8]. The absence of *A. phagocytophilum *in non-blood fed larvae supports the assumption that vertical transmission, i.e. transovum or transovarial transmission from mother tick to her offspring, is of no or little importance for the maintenance of this agent. Most larvae caught by blanket-dragging have generally not yet ingested blood from a vertebrate host. This explains why the larvae were uninfected. Mammals such as rodents and shrews are believed to be important reservoirs and therefore they primarily infect larvae that are ingesting blood [6,7,9-11]. A common feature of *Anaplasma *spp. is that transstadial transmission (from larva to nymph to adult) is ineffective [7,10]. Our findings of *A. phagocytophilum *in the nymphs only supports the view that any potential transstadial transmission from nymph to adult is infrequent in the present study areas. A likely reason for the finding of infected nymphs but uninfected adult ticks is that the majority of the former had fed as larvae on reservoir-competent small mammals, whereas the adult ticks had fed as nymphs on non-reservoir-competent larger mammals, possibly roe deer.

Our findings also support results showing relatively high seroprevalence rates in humans; antibodies reacting to the HGE agent have been found among 28% of the residents in an endemic area in Sweden [16].

The real-time PCR detects a conserved region of the *A. phagocytophilum msp*2 gene that is suggested to be unique to *Anaplasma *species [25,26]. The PCR is designed specifically to amplify the gene in the *A. phagocytophilum *genome, the consequence being that it is not appropriate for detection of *Anaplasma ovis, A. marginale *and *A. centralis *[25]. In our study, the high specificity of the PCR assay for *A. phagocytophilum *was demonstrated by sequencing the amplified product of six positive samples, which only revealed sequences consistent with *A. phagocytophilum*. The 928 and 151 bp-long fragments (16 S rRNA), and sequencing of these fragments of which 850 bp and 90 bp were complete, did not reveal any intraspecies genetic variability, as has been detected in *A. phagocytophilum *from Bavaria, Germany [27]. However, longer fragments and sequencing of other genes may be necessary to assess intraspecies genetic differences in this bacterium.

Only one *Rickettsia *species, namely the spotted fever rickettsia *R. helvetica*, was found in the investigated ticks. The infection rates in larvae and nymphs were similar, while that in adult ticks was significantly lower. These findings are in accordance with results from previous studies in Europe, including Swedish investigations [19,28-32]. *I. ricinus *is the major reservoir host for *R. helvetica; *birds and large mammals may be important for the geographic dispersion of infected ticks, while small mammals may be important for the geographic spread of both rickettsiae and *R. helvetica-*infected ticks [21,33].

The tick infection rates in the different areas should be considered as preliminary; because the samples were pooled, the prevalences can only be roughly estimated. It was surprising that all pools from Bogesund were *Rickettsia *negative, while all pools from Västerås were negative for both *Anaplasma *and *Rickettsia*. Five samples containing a total of 60 nymphs were positive for both agents. *I. ricinus *is a three-host tick and may therefore acquire more than one pathogen species from a different or the same reservoir individual. Moreover, the present study shows that ticks in Sweden, in addition to well-known pathogens such as *Borrelia burgdorferi *s. l. and the TBE virus, also harbour *R. helvetica *and *A. phagocytophilum *and that clinicians should be aware of the fact that different potentially human pathogenic tick-borne bacteria are prevalent in Sweden and may affect the clinical picture. The low number of reported human cases due to *A. phagocytophilum *or *R. helvetica *may possibly be explained by the presence of low virulence strains and/or inadequate diagnoses, and for the moment this finding does not seem to be in accordance with the usual incidence of tick bites and the prevalence of these agents in ticks.

## Conclusions

*A. phagocytophilum *and/or *R. helvetica *are frequently found in *I. ricinus-*infested areas in Sweden. These pathogenic bacteria are a potential threat to public and animal health in areas where *I. ricinus *is abundant, but additional areas need to be examined to provide a more reliable picture of the actual dispersion.

## Materials and methods

### Collection of ticks

**Figure 1:**
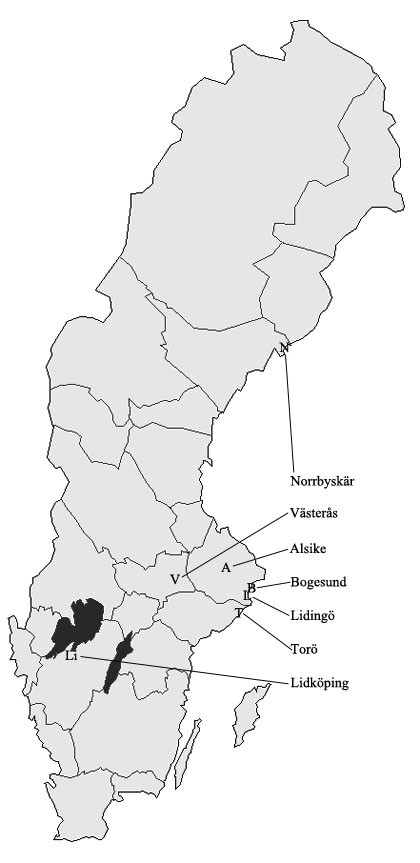
**Location of the seven study areas in which ticks were collected**. Lidingö (L), Alsike (A), Torö (T), Bogesund (B), Västerås (V), Lidköping (Li), Norrbyskär (N).

During April-September 2007, drag sampling using a white flannel cloth of surface area 1 m^2 ^was used at irregular intervals to collect host-seeking ticks from the ground vegetation at 7 locations in central and southern Sweden (Figure 1). The cloth was drawn along the ground for 10 m, flipped, and all ticks were then removed and placed in separate tubes for nymphs, adult females, and adult males. The ticks were kept alive at high humidity (80-90% RH) and +4 to +8°C for a maximum of one week, and identified with regard to developmental stage and species using a dissecting microscope (Leica M5/M8). All 1245 ticks collected belonged to *I. ricinus*. A total of 204 larvae, 963 nymphs and 78 adults (38 males and 40 females) were analysed further. The pooled samples, containing 1-29 tick specimens, were stored in snap-lid tubes and frozen at -70°C. The number of pools was 22 for larvae, 91 for nymphs and 26 for adults (13 with males and 13 with female ticks). The average number of individual ticks in each tube was 9.3 for larvae, 10.5 for nymphs and 3.4 for adults.

### DNA extraction

The ticks were disinfected in 70% ethanol for 10 minutes, rinsed with sterilized distilled water, put in a tube and homogenized in 400 μL sodium phosphate-buffered (100 mM; pH 7.2) isotonic (0.9% NaCl, w/v) saline. RNA and DNA were extracted from the homogenate from each pool using 800 μL Tripure reagent (Roche Diagnostics) and 2 μL (20 mg/mL) glycogen (Invitrogen, Carlsbad, CA) protocol with chloroform. After centrifugation, the lower phase was used for DNA isolation. DNA was precipitated after addition of 300 μL 100% ethanol, washed with 800 μL 0.1 M sodium citrate in 10% ethanol, centrifuged, air dried and the pellet was stored at -20°C for later analyses

### Quantitative Real-time PCR

All pooled samples were analysed individually. The pellet was dissolved in 50 μL 8 mM NaOH + 5 μL 0.1 M HEPES buffer (Roche Diagnostics) and assayed for detection of *A. phagycotophilum *using a real-time PCR with the probe and primers specific to the *msp*2 gene, as previously described [26]. *Rickettsia *spp. was detected using a genus-specific real-time PCR with the probe and primers targeting the *glt*A gene, as described previously (Table 5) [34].

**Table 5:** Details of selected primers and probe used to amplify *Anaplasma *(msp2, 16 S RNA) and *Rickettsia *genes (*omp*B,17 kDa, *glt*A).

Primer	Gene	Nucleotide sequences (5' to 3')	Product size (bp)
Apmsp2f	*msp2*	ATG-GAA-GGT-AGT-GTT-GGT-TAT-GGT-ATT	77
Apmsp2r		TAC-GAG-CGC-TTC--AAG-ACC-AA	
Apmsp2p		TGG-TGC-CAG-GGT-TGA-GCT-TGA-GAT-TG	probe
GER-3	16 S RNA	TAG-ATC-CTT-CTT-AAC-GGA-AGG-GCG	151
GER-4		AAG-TGC-CCG-GCT-TAA-CCC-GCT-GGC	
EE-1	16 S RNA	TCC-TGG-CTC-AGA-ACG-AAC-GCT-GGC-GGC	928
EE-2		AGT-CAC-TGA-CCC-AAC-CTT-AAA-TGG-CTG	
EE-3		GTC-GAA-CGG-ATT-ATT-CTT-TAT-AGC-TTG-C	
EE-4		CCC-TTC-CGT-TAA-GAA-GGA-TCT-AAT-CTC-C	
OMP B-IF	*omp *B	CCA-ATG-GCA-GGA-CTT-AGC-TAC-T	257
OMP B-IR		AGG-CTG-GCT-GAT-ACA-CGG-AGT-AA	
17 SFG-F	17 kDa	GCT-CTTGCA-ACT-TCT-ATG-TT	431
17 SFG-R		CAT-TGT-TCG-TCA-GGT-TGG-CG	
SFG-CS-F	*gltA*	TGC-CAA-ATG-TTC-ACG-GTA-CTT-T	74
SFG-CSR		CAC-AAT-GGA-AAG-AAA-TGC-ACG-A	
SFG-CS-P		TGC-AAT-AGC-AAG-AAC-CGT-AGG-CTG-GAT-G	probe

As a standard, the amplified 77 and 74 bp fragments obtained in real-time PCR of *A. phagocytophilum *and *R. helvetica*, respectively, were ligated into a PCR 4-TOPO vector and transformed into One Shot TOP10 chemically competent *Escherichia coli *following the manufacturer's instructions (TOPO TA Cloning^® ^Kit for Sequencing, Invitrogen). Tenfold serial dilutions of extracted plasmids were used to establish standard curves for the PCR runs. The quantification was linear over a range of 10 to 10^8 ^copies, and the detection limit was shown to be 1-10 copies per reaction. Both real-time PCR assays were performed in a Rotor-Gene 3000 (Corbett Research, Sydney, Australia) using LC Taqman Master kit (Roche).

### *Anaplasma *and *Rickettsia *species identification and genotyping

For *Anaplasma *sp., samples positive in real-time PCR were further analysed using two PCR assays that amplify partial sequences of the 16 S rRNA gene (151 and 928 bp), as previously reported (Table 5) [35,36]. As a positive control in the conventional PCR, we used DNA extracted from *A. phagocytophilum *(provided by the Section of Zoonotic Ecology and Epidemiology, Kalmar University, Kalmar, Sweden).

For *Rickettsia *spp., the positive samples were further analysed using conventional and nested PCR assays that amplify partial sequences of the 17-kDa and *omp*B genes, with the expected fragment lengths of 431 and 266 bp, as previously described [31,37]. Conventional and nested PCR were performed in a DNA thermal cycler (GeneAmp PCR System 9700 (PE Applied BioSystems), and expected fragment sizes were confirmed using gel electrophoresis (2% agarose, 1% ethidiumbromide). Confirmation of fragment size was based on a standard DNA molecular weight marker (Invitrogen). As a negative control, sterile water was included in each amplification trial. Purified DNA of *R. helvetica *originally isolated from a *I. ricinus *tick was used in these assays as the positive control (Table 5) [19].

Direct cycle sequencing analysis of both strands of amplicons was performed using an automatic Hitachi 3100 Avant Plus Genetic Analyzer (Applied Biosystems, Tokyo, Japan). For species identification, pair-wise similarities to and differences from other rickettsiae in the spotted fever group were examined using Blast analysis. Multiple sequence alignments were conducted using BioEdit version 7.0.9 and ClustalW.

### Statistical analysis

Odds ratio procedures (OR), Fisher's exact test and *x*^*2*^-test, were used to compare the proportions and a *P *value < 0.05 was considered statistically significant. Statistical analyses were conducted using Predictive Analytics Software (PASW^®^) Statistics 18.

## Competing interests

The authors declare that they have no competing interests.

## Authors' contributions

TGJ, JP, KF and KN conceived and designed the experiments; KS, TGJ, JP, KN and KF performed the experiments and analysed the data. All authors read and approved the final manuscript.
